# Is radial extracorporeal shock wave therapy combined with a specific rehabilitation program (rESWT + RP) more effective than sham-rESWT + RP for acute hamstring muscle complex injury type 3b in athletes? Study protocol for a prospective, randomized, double-blind, sham-controlled single centre trial

**DOI:** 10.1186/s13018-019-1283-x

**Published:** 2019-07-23

**Authors:** Javier Crupnik, Santiago Silveti, Natalia Wajnstein, Alejandro Rolon, Alisa Vollhardt, Peter Stiller, Christoph Schmitz

**Affiliations:** 1 KinEf Kinesiología Deportiva, Buenos Aires, Argentina; 2 IMAXE Diagnostico X Imagenes, Buenos Aires, Argentina; 3 Extracorporeal Shock Wave Research Unit, Chair of Neuroanatomy, Institute of Anatomy, Faculty of Medicine, LMU Munich, 80336 Munich, Germany; 4 Department of General Medicine, Clinic Lechhausen, Augsburg, Germany; 5 Medical Team, FC Augsburg 1907 Football Club, Augsburg, Germany

**Keywords:** Acute hamstring muscle complex injury 3b, Athletes, ESWT, Radial extracorporeal shock wave therapy, rESWT, Rehabilitation

## Abstract

**Background:**

Acute injuries of the hamstring muscle complex (HMC) type 3b (interfascicle/bundle-tear) are frequently observed in various sports disciplines both in elite and recreational sport. The treatment of choice of acute HMC injuries type 3b is a progressive physiotherapeutic exercise programme. Besides this, there is currently only insufficient scientific evidence to support other treatment methods, including local infiltrations and injections of platelet-rich-plasma. Very recently, it was demonstrated that extracorporeal shock wave therapy (ESWT) may accelerate regeneration after acute skeletal muscle injury. The aim of the present study is to test the hypothesis that the combination of radial ESWT (rESWT) and a specific rehabilitation program (RP) is effective and safe in treatment of acute HMC injury type 3b in athletes, and is statistically significantly more effective than the combination of sham-rESWT and RP.

**Methods:**

We will perform a double blind, randomized, sham-controlled clinical trial at the clinic KinEf Kinesiología Deportiva, Ciudad Autónoma de Buenos Aires, Argentina. Forty patients with acute HMC injury type 3b will be randomly allocated to receive either rESWT (nine rESWT sessions; three sessions per week; 2500 radial extracorporeal shock waves (rESWs) per session; energy density depending on what the patient tolerates) or sham-rESWT. In addition, all patients will receive a specific rehabilitation program that will last for 8 weeks. The primary outcome measure will be the individual time (days) necessary to return to play. Secondary outcomes will include the presence or absence of reinjury during a time period of 6 months after inclusion into the study.

**Discussion:**

Because of the lack of adequate treatment options for acute HMC injury type 3b in athletes and particularly the high reinjury rate, we hypothesize that the results of this trial will be of importance and have impact on clinical practice.

**Trial registration:**

ClinicalTrials.gov ID NCT03473899. Registered March 22, 2018.

## Background

Acute injuries of the hamstring muscle complex (HMC) are frequently observed in various sports disciplines both in elite and recreational sport [1–3], and are the most common injury in soccer (e.g., [4, 5]). Despite intensive research into prevention and management of acute HMC injury during the last decade, epidemiological data show no decline in injury and reinjury rates [6]. In this regard, 374 Danish elite soccer players were prospectively observed during a 12-month period, during which 46 first-time and eight recurrent HMC injuries were documented (incidence rates: 12.3% [first-time injuries] and 2% [recurrent injuries]) [7]. Statistically significantly more players experienced a first-time acute HMC injury during a match than during training in this study [7]. Moreover, among 32 players who suffered from acute HMC injury in a period of 12 months before the study, eight players incurred an injury that fulfilled the criteria for a recurrent injury (incidence 25%) [7]. Approximately two-thirds of the first-time injuries were categorized as moderate, with time to return to play between 8 and 28 days [7].

Anatomical and functional aspects of the HMC predispose it to injury, including the fact that the muscles cross two joints and undergo eccentric contraction during the gait and running cycle [8]. Acute HMC injury typically occurs through an eccentric mechanism at the terminal stages of the swing phase of running [9]. The long head of the biceps femoris (LHBF) muscle is most commonly affected, and within the LHBF muscle, the proximal myotendinous junction and proximal locations are most commonly affected [10].

The diagnosis of acute HMC injury is based on the presence of acute-onset pain in the posterior thigh, and presence of the triad of pain on contraction, stretching, and palpation [11]). Imaging has a role in confirming the site of injury and characterizing its extent, providing some prognostic information and helping plan treatment [8]. In this regard, both magnetic resonance imaging (MRI) and ultrasonography (US) have been shown to be effective for identification of hamstring strains and tendinopathy (e.g., [12–15]). Both MRI and US provide detailed information about the HMC with respect to localization and characterization of injury [13]. In a recent systematic review [16], several clinical, MRI, and US determinants were determined that are associated with a longer recovery time in nonoperative management of acute HMC injury (summarized in Table 1). However, it is important to realize that for an individual HMC injury, none of these MRI and US determinants show a direct correlation with the time to return to play [14, 15]. Accordingly, the prognosis of HMC injuries should not be guided by imaging findings alone [14].

**Table 1:** Determinants having an effect on the time to return to play after hamstring muscle complex injury in athletes (according to [16])

Clinical determinants	MRI determinants	US determinants
• Stretching-type injuries	• Positive findings	• Large cross-sectional area
• Recreational-level sports	• Higher grade of injury	• Injury outside the musculotendinous junction
• Structural versus functional injuries	• Muscle involvement > 75%	• Hematoma
• Greater range of motion deficit with the hip flexed at 90°	• Complete transection	• Structural injury
• Time to first consultation > 1 week	• Retraction	• Injury involving the biceps femoris
• Increased pain on the visual analog scale	• Central tendon disruption of the biceps femoris	
• > 1 day to be able to walk pain free after the injury	• Proximal tendon involvement	
	• Shorter distance to the ischial tuberosity	
	• Length of the hamstring injury	
	• Depth, volume, and large cross-sectional area	

*MRI* magnetic resonance imaging, *US* ultrasonography

According to [1], muscle injuries in sports (including acute HMC injuries) can be classified as shown in Table 2. This classification has important implications for treatment and prognosis (i.e., time to return to play).

**Table 2:** Classification of muscle injuries in sports (according to [1])

A: indirect muscle injuries	
Type 1 and 2: functional muscle disorder	
Type 1: overexertion-related muscle disorder	
Type 1a: fatigue-induced muscle disorders	
Type 1b: delayed-onset muscle soreness	
Type 2: neuromuscular muscle disorder	
Type 2a: spine-related neuromuscular disorders	
Type 2b: muscle-related neuromuscular disorders	
Type 3 and 4: structural muscle injury	
Type 3: partial muscle tears	
Type 3a: minor partial muscle tear (Y 5 mm; intrafascicle/bundletear)	
Type 3b: moderate partial muscle tear (> 5 mm; interfascicle/bundle-tear	
Type 4: subtotal/complete muscle tear or tendinous avulsion	
B: direct muscle injuries	
Contusion	
Laceration	

Acute HMC injuries type 4 (i.e., subtotal or complete muscle tear or tendinous avulsion according to [1]) require early surgical repair (e.g., [17–19]). However, acute HMC injuries type IV are rare [11].

The treatment of choice of acute HMC injuries type 3a and 3b is a progressive physiotherapeutic exercise programme (e.g., [11, 12, 20, 21]). Besides this, there is currently only insufficient scientific evidence to support other treatment methods, including local infiltrations as recommended in [1] (c.f. [11, 22]). In particular, injections of platelet-rich plasma (PRP) showed no effect when compared to control (e.g., [23–25]). A very prominent study that was recently published in *The New England Journal of Medicine* demonstrated no benefit for intramuscular PRP injections, as compared with placebo injections, in patients with acute hamstring injuries [26].

It is of note that another study that was published very recently in the *The New England Journal of Medicine* demonstrated the negative clinical consequences of protracted immobilization after an acute muscle injury type 3b in recreational sports [27]. Starting rehabilitation 2 days after injury rather than waiting for 9 days shortened the interval from injury to pain-free recovery and the time to return to play by approximately 3 weeks without any significant increase in the risk of reinjury [27]. The authors of this study concluded that the observed difference supports the importance of early loading of injured musculotendinous tissue [27].

According to [1], acute muscle injuries type 3a and 3b have different time frames for recovery and return to play, with optimal treatment between 10 and 14 days in case of type 3a and on average approximately 6 weeks in case of type 3b. However, particularly in case of acute HMC injury type 3b, there is considerable interindividual variability in the time frame for return to play, which varied between 14 and 105 days in [26] and between 30 and 233 days in [27]. These data are in line with an earlier report on time frames for return to play after posterior thigh muscle injury in elite soccer players [28] but are considerably longer than the time frame of 25–35 days for return to play after type 3b injuries described in [12]. One reason for this discrepancy might be that the latter report [12] was not restricted to hamstring muscle injuries (as in [26]) or to injuries of the thigh muscles and calf muscles (as in [27]).

Most importantly, particularly the high reinjury rate of acute HMC injury suggests that commonly utilized rehabilitation programs may be inadequate at resolving possible muscular weakness, reduced tissue extensibility, and/or altered movement patterns associated with the injury [29]. Accordingly, there is a need for developing innovative treatment options particularly for acute HMC injury type 3b.

Very recently, it was demonstrated that extracorporeal shock wave therapy (ESWT) may accelerate regeneration after acute skeletal muscle injury [30]. The use of extracorporeal shock waves in medicine was first reported over 30 years ago as a treatment for kidney stones [31], and is commonly referred to as “extracorporeal shock wave lithotripsy,” or “ESWL” [32]. Extracorporeal shock waves are also used as a treatment for musculoskeletal conditions such as plantar heel pain (reviewed in, e.g., [33, 34]) and boney non-union (reviewed in, e.g., [35]), and is commonly referred to as “extracorporeal shock wave therapy” (ESWT) to differentiate from ESWL [33].

**Fig. 1:**
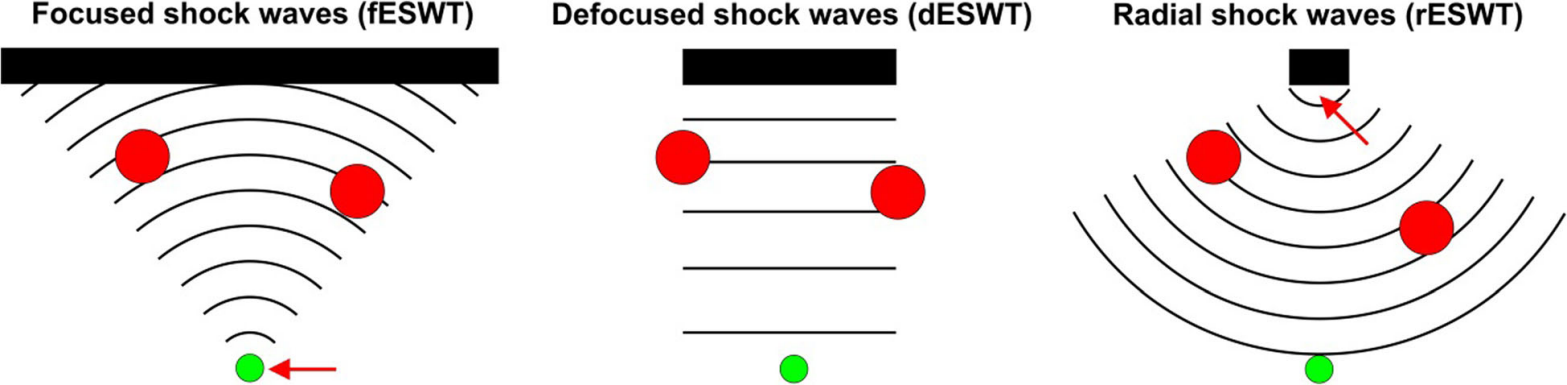
Working principle of focused (on the left), defocused (in the middle), and radial (on the right) extracorporeal shock wave technology. In case of focused shock waves, single acoustic pulses are generated either with a spark-gap (electrohydraulic principle), a technology similar to a loudspeaker (electromagnetic principle), or piezocrystals (piezoelectric principle) (black bars represent shock wave generators; details are provided in Fig. 2). By means of reflectors of certain shape and/or the use of acoustic lenses, the acoustic pulses are converted into a focused acoustic pressure wave/shock wave with a point of highest pressure (red arrow) at the desired target (green dot) within pathological tissue. By changing the shape of the reflector (and/or the acoustic lens), the acoustic waves emitted from a focused shock wave generator can be converted into a slightly convergent, parallel, or even divergent acoustic pressure wave/shock wave (“defocused shock wave”). In case of radial shock waves, a projectile is fired within a guiding tube that strikes a metal applicator placed on the patient’s skin. The projectile generates stress waves in the applicator that transmit pressure waves into tissue. The point of highest pressure is found at the tip of the applicator. It is of note that any disturbance in the pathway of the acoustic pulses between a focused shock wave source and the target within tissue (such as bone, calcifications, etc.; red dots in the figures) may result in some parts of the acoustic pulse not reaching the target and, thus, weakening the shock wave energy (i.e., the energy density) at the target. The same disturbances would not impact the energy of radial shock waves at the target (for defocused shock waves, it is unknown to what extent they are weakened by disturbance in the pathway of the acoustic pulses between the shock wave source and the target within tissue). This is most probably the reason why in muscle tissue, the energy of focused shock waves was found to be decreased by > 50% compared to measurements in water, whereas for radial shock waves measurements in muscle tissue and water were consistent [36]

**Fig. 2:**
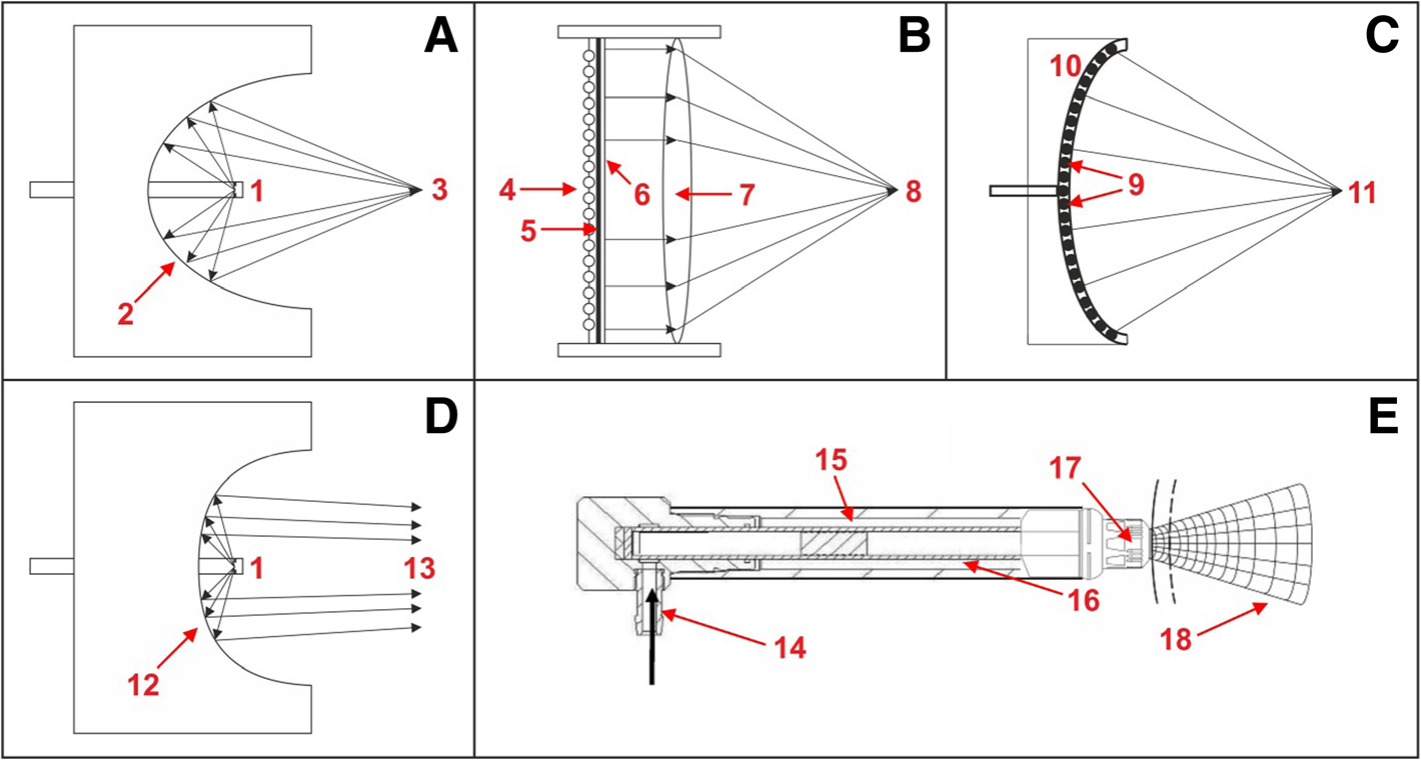
Schematic representation of the mode of operation of focused (**a**–**c**), defocused (**d**), and radial (**e**) extracorporeal shock wave generators. **a** Electrohydraulic principle (fESWT): a high-voltage discharges rapidly across two electrode tips (spark-gap) (1) that are positioned in water. The spark-gap serves as the first focal point (1). The heat generated by this process vaporizes the surrounding water. This generates a gas bubble centered on the first focal point, with the gas bubble being filled with water vapor and plasma. The result of the very rapid expansion of this bubble is a sonic pulse, and the subsequent implosion of this bubble causes a reverse pulse, manifesting a shock wave. By means of reflectors of certain shape (2), this shock wave can be converted into a convergent/focused acoustic pressure wave/shock wave with a point of highest pressure at the second focal point (3). **b** Electromagnetic principle (fESWT): a strong, variable magnetic field is generated by passing a high electric current through a coil (4). This causes a high current in an opposed metal membrane (5), which causes an adjacent membrane (6) with surrounding liquid to be forced rapidly away. Because the adjacent membrane is highly conductive, it is forced away so rapidly that the compression of the surrounding liquid generates a shock wave within the liquid. By means of an acoustic lens (7) of certain shape, this shock wave can be converted into a convergent/focused acoustic pressure wave/shock wave with a point of highest pressure at a focal point (8). **c** Piezoelectric principle (fESWT): a large number of piezocrystals (9) are mounted in a bowl-shaped device (10); the number of piezocrystals can vary from a few to several thousands (typically between 1000 and 2000). When applying a rapid electrical discharge, the piezocrystals react with a deformation (contraction and expansion), which is known as the piezoelectric effect. This induces an acoustic pressure pulse in the surrounding water that can steep into a shock wave. Because of the design of the bowl-shaped device, an acoustic pressure wave/shock wave can emerge with a point of highest pressure at a focal point (11). **d** Defocused principle (shown here for the electrohydraulic principle). By changing the shape of the reflector (12), the shock wave emitted from the first focal point is converted into a slightly convergent, parallel, or even divergent acoustic pressure wave/shock wave (“defocused shock wave”) (13). **e** Ballistic principle (rESWT): compressed air (pneumatic principle; 14) or a magnetic field (not shown) is used to fire a projectile (15) within a guiding tube (16) that strikes a metal applicator (17) placed on the patient’s skin. The projectile generates stress waves in the applicator that transmit pressure waves into tissue (18)

There are three different types of extracorporeal shock waves that could be used in ESWT for acute HMC injury type 3b, focused, defocused, and radial (Fig. 1), and several modes of operation of focused, defocused, and radial extracorporeal shock wave generators (Fig. 2).

To our knowledge, randomized controlled trials (RCTs) testing efficacy and safety of ESWT for acute HMC injury type 3b have not yet been published. In contrast, ESWT has become an established treatment modality for various musculoskeletal conditions such as calcifying tendonitis of the shoulder, tennis elbow, and plantar fasciopathy, to mention only a few (details can be found in [34]). Among the 44 RCTs on rESWT currently listed in the PEDro database [37, 38] (status of September 09, 2017), 29 (66%) were performed with the rESWT device Swiss DolorClast (Electro Medical Systems, Nyon, Switzerland).

Some of us (J.C., P.S. and C.S.) have extensive practical experience with rESWT for various musculoskeletal conditions using the Swiss DolorClast. Most importantly, we have already gained practical experience with rESWT for acute HMC injury type 3b in athletes. One of our (P.S. and C.S.) most prominent patients was a professional soccer player at a European top club (regularly playing in the UEFA Champions League) who incurred a HMC injury type 3b and returned to play (full 90-min competitive match) 35 days later. In the aforementioned studies published in the *New England Journal of Medicine* [26, 27], the cumulative probability of resumptions of sports activity on day 35 after acute HMC injury type 3b in professional soccer players [26] or recreational athletes [27] was only respectively 20% [26] or 5% [27] after treatment with a rehabilitation program.

Considering the limited evidence of efficacy and safety of rESWT for acute HMC injury type 3b, further research is needed to support the use of rESWT for this condition. Taking into account the proven efficacy and safety of rESWT using the Swiss DolorClast for treating musculoskeletal conditions [34], the widespread use of the Swiss DolorClast based on its proven efficacy and safety, and our own very promising pilot data of rESWT using the Swiss DolorClast for treating acute HMC injury type 3b in athletes, it is reasonable to hypothesize that (i) the combination of rESWT and a specific rehabilitation program is effective and safe in treatment of acute HMC injury type 3b, (ii) this combination therapy is statistically significantly more effective than the combination of sham-rESWT and the same specific rehabilitation program, and (iii) this combination therapy will gain widespread acceptance as soon as effectiveness and safety will be demonstrated in a randomized controlled trial. This is the main purpose of the proposed study.

## Methods

### Aims

1. To determine the efficacy and safety of radial extracorporeal shock wave therapy combined with a specific rehabilitation program (rESWT + RP) compared with sham-rESWT + RP in treatment of acute HMC injury type 3b.
2. To determine the individual and mean time to return to play after treating acute HMC injury type 3b with respectively rESWT + RP or sham-rESWT + RP.
3. To determine the incidence of reinjury during a period of 6 months after return to play following treatment of acute HMC injury type 3b with respectively rESWT + RP or sham-rESWT + RP.
4. To evaluate patient’s pain score during respectively rESWT or sham-rESWT for acute HMC injury Type 3b using the Visual Analogue Scale (VAS) score.
5. To evaluate patient’s satisfaction at six months after the end of treatment.

### Study design

This is a randomized controlled trial (RCT) on rESWT + RP vs. sham-rESWT + RP, with blinding of patients and evaluators/assessors, but without blinding of the therapist who will perform the rESWT. All the patients will be recruited from the Club Deportivo UAI Urquiza (Villa Lynch, Province Buenos Aires, Argentina). Officials of the Club Deportivo UAI Urquiza will be instructed that athletes who experience sudden, sharp pain in the posterior aspect of the thigh during training or competition shall immediately stop activity. These athletes will then be evaluated regarding the presence of the inclusion criteria of this study on the day of injury.

**Fig. 3:**
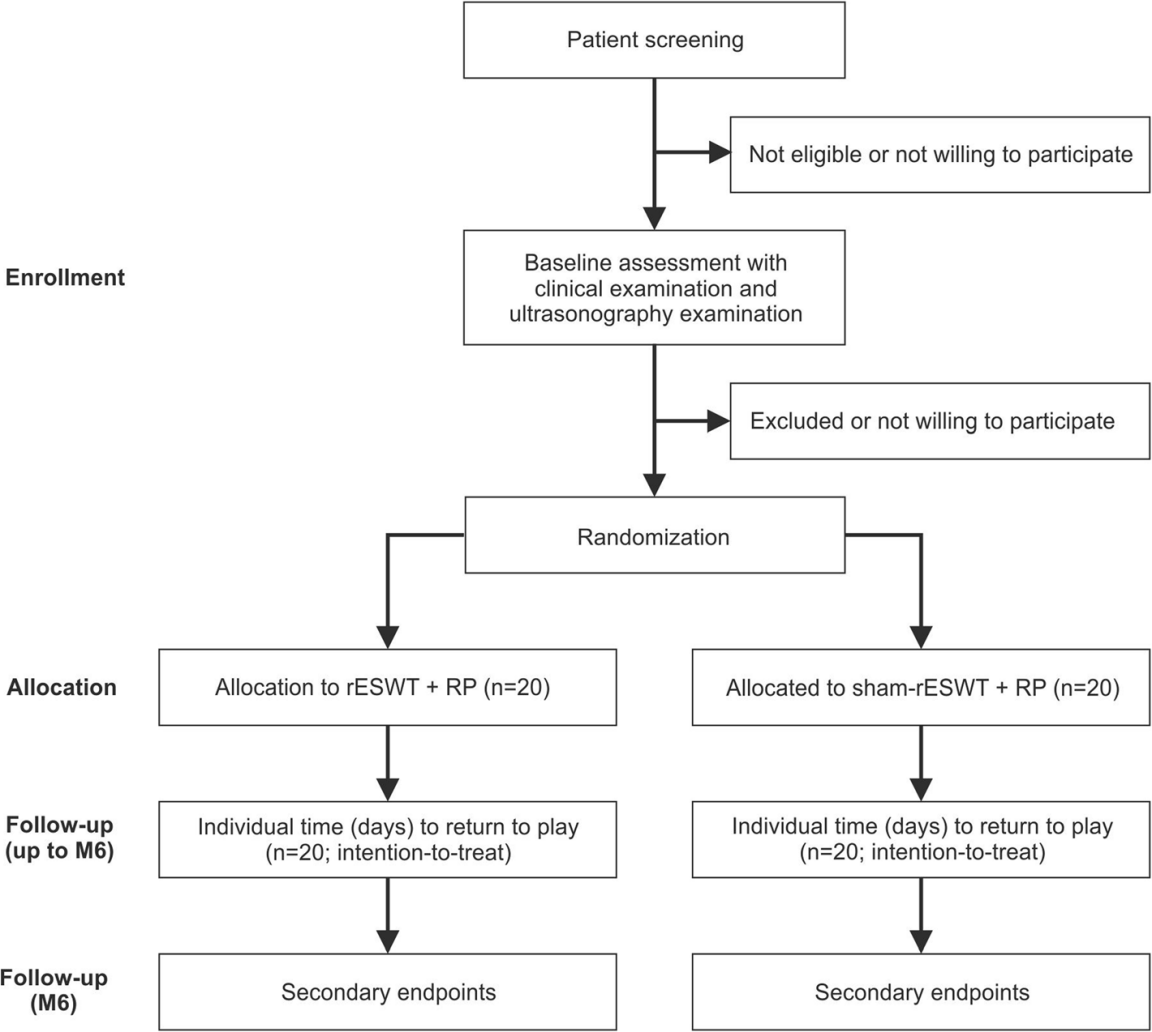
Flow of patients through the present study according to the CONSORT statement [39]

Figure 3 shows the flow of patients through the present study according to the CONSORT statement [39], and Table 3 the schedule of enrollment, interventions, and assessments according to SPIRIT [40].

**Table 3:** Schedule of enrollment, interventions, and assessments during the present study according to SPIRIT [40]

Timepoint	Study period					
	Enrollment/Allocation	Post-allocation			Follow-up	Close-out
	D0, D1, or D2	D0-D3 or D1-D4 or D2-D5	D5, D7, D9, D12, D14, D16, D19, D21, and D23	D5–D60	Between D5 and M6	M6
Enrollment						
Clinical evaluation	X					
Ultrasonographic evaluation	X					
Eligibility screen	X					
Allocation	X					
Interventions						
RICE		X				
rESWT			X			
sham-rESWT			X			
RP				X		
Assessments						
A, G, BMI, S, P, SG	X					
Individual time to return to play					X	
Secondary endpoints						X

*D* day; *M* month; *RICE* rest, ice, compression, and elevation; *rESWT* radial extracorporeal shock wave therapy; *RP* rehabilitation program; *A* age; *G* gender; *BMI* body mass index; *S* sport that is practiced; *P* position in the field; *SG* sporting gesture that caused an injury; *D* day; *M* month

### Ethics

This study has received approval from the local institutional ethics board of the Universidad Abierta Interamericana, Buenos Aires, Argentina (Nr. 0-1027).

### Participants

Adults aged 18–35 years (both female and male) with clinical and ultrasonographic diagnosis of acute HMC injury type 3b are eligible for inclusion.

The inclusion criteria are physical conditions for rehabilitation (i.e., no surgery required), willingness of the patient to participate in the study, written informed consent signed and personally dated by the patient, and no contraindications for rESWT.

The exclusion criteria are children and teenagers below the age of 18, adults aged > 35 years old, patients with clinical and ultrasonographic diagnosis of acute HMC injury type 3b who got injured more than 7 days before potential enrollment into this study, patients with clinical and ultrasonographic diagnosis of acute HMC injury type 3A or type 4, bilateral acute HMC injury (types 3A, 3B, or 4), proven or suspected HMC injury (types 3A, 3B, or 4) of the same lower limb in the time period of 6 months before potential enrollment into this study, muscle injury caused by external impact on the back of the affected thigh (category B according to [1]), surgery on the affected lower limb in the time period of 1 year before potential enrollment into this study, acute or chronic lumbar pathology (because some cases of thigh pain may relate to spinal pathology; c.f. [8]), no willingness of the patient to participate in this study, and/or written informed consent not signed and not personally dated by the patient, and contraindications of rESWT (including treatment of pregnant patients, patients with blood-clotting disorders (including local thrombosis), patients treated with oral anticoagulants, patients with local bacterial and/or viral infections/inflammations, patients with local tumors, and patients treated with local corticosteroid applications in the time period of 6 weeks before the first rESWT session (if applicable)).

### Randomization and blinding

The patients that fulfill the inclusion criteria and do not fulfill any of the exclusion criteria will be randomly allocated to either rESWT + RP (*n* = 20) or sham-rESWT + RP (*n* = 20). Randomization will be performed as described in [41] in a randomized, controlled study on rESWT for Achilles tendinopathy. Specifically, a computerized random-number generator will be used to formulate an allocation schedule. Patients will be randomized to either treatment (rESWT + RP or sham-rESWT + RP), with use of the method of randomly permuted blocks. The randomization scheme will be generated with the use of the website, www.randomization.com. Forty patients will be randomized into five blocks. A medical assistant at the clinic of the Principal Investigator (J.C.) will allocate interventions by means of opaque sealed envelopes that will be marked according to the allocation schedule. The medical assistant will be unaware of the size of the blocks. The randomized intervention assignment as outlined above will be concealed from both patients and health care staff until recruitment will be complete and irrevocable.

Patients and the assessor will be blinded in this study. The assessor is the person who will assess the outcome of treatment during follow up. In this study, the assessor will be a medical doctor affiliated with IMAXE Diagnostico X Images, Ciudad Autónoma de Buenos Aires, Argentina (AR). IMAXE itself is affiliated with the Faculty of Medicine at the Universidad de Buenos Aires, Argentina.

The therapist will not be blinded in this study. This will be done because even when using coded “active” and “sham” handpieces in a study on rESWT, blinding of therapists can only be achieved when another person prepares the device before rESWT or sham-rESWT. This, however, is almost impracticable and has not been done in any of the more than 100 studies on radial and focused ESWT listed in the PEDro database [34]. The solution to this issue is a strict, standardized way of interaction between the therapist and the patients, irrespective of treatment allocation (as mentioned in [42]; c.f. also [43]). This approach will also be applied in the present study. The therapist is the person who will administer either rESWT or sham-rESWT to the patient. In this study, the therapist will be the principal investigator.

### Interventions

All patients will perform a specific rehabilitation program (RP) that will last for 8 weeks, independent of the individual time interval to return to play (in line with [27]). This RP was developed based on recommendations in the literature [44–46]. The key objective of this RP is that after injury, the patient will develop functional, neuromuscular, and biomechanical skills according to the demands of the sport she/he performs, while minimizing the risk of reinjury. Therefore, the proposed RP will take the patient through a combination of low-risk and high-demand movements, based on a systematic process. This process will consist of an orderly sequence of steps or phases—acute phase, subacute/regeneration phase, and functional phase. Each phase will depend on the outcome of the previous phase and will use the individualized response as criterion of progression. The RP will be controlled by the same physiotherapist who will not participate in the inclusion/exclusion process or any subsequent evaluation of the patient.

The goals of the acute phase include to (i) prevent re-rupture at the injured site; (ii) prevent excessive inflammation and formation of scar tissue; (iii) increase tensile strength, adhesion, and elasticity of new granulation tissue; (iv) reduce build-up of interstitial fluid; and (v) detect and treat any lumbopelvic dysfunction. Once a patient will be included in the proposed study, she/he will be instructed to avoid the use of medication and apply the RICE principle (rest, ice, compression, and elevation) three times per day in order to stop the injury-induced bleeding into the muscle tissue and thereby minimize the extent of the injury (see, e.g., [47]).

With regard to the optimum time interval for starting active rehabilitation after acute HMC injury type 3b, some authors recommended immobilization for 3 to 5 days, followed by active mobilization [47]. Other authors pointed out that starting rehabilitation 2 days after injury rather than waiting for 9 days shortened the interval from injury to pain-free recovery and the time to return to play by approximately 3 weeks without any significant increase in the risk of reinjury [27]. However, it is not known whether starting rehabilitation already 2 days after injury has any benefit over starting rehabilitation 5 days after injury. We will therefore follow the recommendation in [47] and progress to the subacute phase after 5 days.

The criterion for progression to the subacute/regeneration phase will be absence of pain 5 days after injury. If the symptoms caused by the injured muscle persist for more than 5 days, we will reconsider the existence of more extensive tissue damage and/or intramuscular hematoma that might require special attention and treatment by an orthopedic surgeon.

The goals of the subacute/regeneration phase include to (i) improve overall core stability; (ii) improve strength and symmetry; (iii) reduce pain during prone isometric, isolated hamstring contractions at 15° knee flexion; (iv) improve hamstring flexibility of both legs; (v) improve hip flexor flexibility of both legs; and (vi) improve neuromuscular control. During the subacute/regeneration phase, the patient will work on both legs daily during a single session. Exercises will be conducted to correct the different risk factors and mechanisms related to the lesion of the hamstring musculature. The exercises will be divided into four groups: core stability and lumbopelvic control, flexibility and neural mobilization, hamstring and gluteal strength, and running technique. In addition, basic aerobic conditioning will start when the patient will be able to perform at least three sessions of the running technique without any discomfort or pain. Three running sessions per week will be performed at the clinic of the principal investigator and will include four sets of 5 min at a low to moderate intensity (individually rated by the patient). Suspension of running sessions will be permitted in the event of discomfort or pain.

The criteria for progression to the functional phase will be no pain in prone position with knee flexed to 15°, no pain during slump test, <  10% asymmetry when in prone position with knee flexed to 15°, <  10% asymmetry during active knee extension test, and < 5° asymmetry in the modified Thomas test.

The goals of the functional phase include to (i) increase the optimum length of the hamstrings, (ii) decrease leg asymmetries in optimum length, (iii) decrease leg asymmetries in concentric hip extension, (iv) decrease leg asymmetries in horizontal force production during running, and (v) improve torsional capabilities. The functional phase will comprise daily exercises, with three sessions per week at the clinic of the principal investigator (every other day) and the remaining sessions at the club or at home. The exercises will comprise the following: core stability and lumbopelvic control, flexibility and neural mobilization, hamstring and gluteal strength, plyometric training, and running technique. During the functional phase, the running session will consist of two sets of 10 min at moderate to high intensity (individually rated by the patient). Suspension of running sessions will be permitted in the event of discomfort or pain.

The criteria for return to play will be (according to [48]) absence of pain on palpation, absence of pain during flexibility testing (active knee extension test and passive straight leg raise test), absence of pain during strength testing (isometric force test), absence of pain during and after functional testing (repeated sprint ability test and single leg bridge), similar hamstring flexibility, psychological readiness/athlete confidence, and clearance by the medical staff. The quantity and quality of the supervised rehabilitation sessions at home or the sports club will be documented.

Patients in the rESWT group will receive the specific rehabilitation program as outlined above, and rESWT as follows: nine rESWT sessions; three sessions per week (interval between sessions: two or three days); rESWT device: Swiss DolorClast (Electro Medical Systems, Nyon Switzerland), EvoBlue handpiece, 15 mm applicator; 2500 rESWs per session, with energy density between 0.12 and 0.16 mJ/mm^2^ (achieved by operating the Swiss DolorClast at air pressure between three and four bar), depending on what the patient tolerates; 15 rESWs per second, resulting in treatment time between 3 and 5 min per session; application of rESWs in prone position, with the patient lying on an examination table; exact location of the application of rESWs determined by clinical and ultrasonographic examinations; treatment of both the side of injury and the entire affected muscle (from distal to proximal in order to relax the affected muscle); application of rESWs in sagittal (dorsal—ventral) direction; and no use of local anesthesia.

Patients in the sham-rESWT group will receive the specific rehabilitation program as outlined above, and sham-rESWT as outlined above, with a specially designed sham EvoBlue handpiece that looks and sounds like the EvoBlue handpiece of the Swiss DolorClast, but does not generate radial shock waves. This is achieved by blocking the projectile (“15” in Fig. 1) shortly before it strikes the metal applicator (“17” in Fig. 2). The sham EvoBlue handpiece will not emit any radial shock wave energy.

### Study treatment and visits

All patients will perform RP that will last for 8 weeks, independent of the individual time interval to return to play. The RP will start with a first visit to the clinic of the principal investigator during which clinical and ultrasonographic diagnosis will be performed. This first visit may take place on the day of injury (D0) or the first day (D1) or second day (D2) after injury (the sooner the better).

After the first visit, an individual number of days will follow until the fifth day after injury (D5) will be reached (acute phase). During this time, the patient will apply the RICE principle (rest, ice, compression, and elevation). Visits to the clinic of the principal investigator may be scheduled during this time but are not mandatory.

On D5, the subacute/regeneration phase of the RP will start, with three visits per week to the clinic of the principal investigator. The exact time for progression from the subacute/regeneration phase to the functional phase of the RP will be individually determined, depending on whether the criteria for progression will be fulfilled. During the functional phase, there will also be three visits per week to the clinic of the principal investigator.

Study treatments (rESWT or sham-rESWT) will start on D5. Each patient will be treated with nine sessions of rESWT or sham-rESWT, with three sessions per week. Accordingly, study treatments may take place at D5, D7, D9, D12, D14, D16, D19, D21, and D23.

Six months after inclusion into the study, there will be a separate visit for evaluating patient’s satisfaction with the treatment outcome.

The time interval necessary for reaching return to play will be as follows: based on our experience, we expect that approximately 75% of the patients treated with rESWT + RP will reach return to play within 5 weeks after D0. Furthermore, we expect that only approximately 25% of the patients treated with sham-rESWT + RP will reach return to play within 5 weeks after D0.

### Outcome measurements and assessments

The primary clinical outcome will be the individual time (days) necessary to return to play. Individual treatment success is defined as the possibility to return to play with the following criteria fulfilled (according to [48]): absence of pain on palpation, absence of pain during flexibility testing (active knee extension test and passive straight leg raise test), absence of pain during strength testing (isometric force test), absence of pain during and after functional testing (repeated sprint ability test and single leg bridge), similar hamstring flexibility, psychological readiness/athlete confidence, and medical staff clearance.

Secondary clinical outcomes will be individual patient’s satisfaction at 6 months after inclusion into the study (using a scale ranging from 0 (maximum dissatisfaction) to 10 (maximum satisfaction)), and presence or absence of reinjury during a time period of 6 months after inclusion into the study (defined as sudden, sharp pain in the posterior aspect of the thigh that was initially injured, accompanied by the same objective criteria initially used for the diagnosis of acute HMC injury type 3b).

In addition to primary and secondary clinical outcomes, the following parameters will be evaluated and reported: patient’s sex, age, weight, height, and body mass index; the interval between injury and the first treatment (in days); and patient’s individual training load (number of training sessions per week; duration of training sessions).

Note that we will document the anatomical location of the injury. However, in line with the literature [26–28], we will not perform a sub-analysis with respect to a possible correlation between the results of clinical intervention and the anatomical location of the injury.

### Sample size

In the aforementioned studies [26, 27]), the cumulative probability of resumptions of sports activity on day 35 after acute HMC injury type 3b in professional soccer players [26] or recreational athletes [27] was only respectively 20% [26] or 5% [27] after treatment with a rehabilitation program.

On this basis, we performed a power analysis for a percentage of 25% as well as for various other percentages (ranging between 10 and 99.9%) of patients with treatment success when treated with sham-rESWT + RP (*n* = 20), accounting for a two-sided confidence interval of 95% (and, thus, a type-1 error rate of 5%) and a percentage of patients with treatment success when treated with rESWT + RP (*n* = 20) of 75%. Calculations were performed using the software, Open Source Epidemiologic Statistics for Public Health (www.openepi.com). Furthermore, we calculated the minimum sample size in both groups (rESWT + RP, sham-rESWT + RP) that would be necessary for detecting a difference in treatment success between the patients treated with rESWT + RP and the patients treated with sham-rESWT + RP accounting for a two-sided confidence interval of 95% and a power of 0.8. Calculations were also performed using the software, Open Source Epidemiologic Statistics for Public Health (www.openepi.com). The results are summarized in Tables 4 and 5.

**Table 4:** Power for the proposed RCT on rESWT + RP for acute HMC injury type 3b, accounting for a two-sided confidence interval of 95% and a percentage of patients with treatment success when treated with rESWT + RP of 75%

Percent of patients treated with sham-rESWT + RP with treatment success (%)	Power based on normal approximation (%)	Power based on normal approximation with continuity correction (%)
99.9	67.4	44.8
90	23.4	10.3
80	5.3	5.3
70	5.0	5.0
60	16.9	8.0
50	36.8	23.6
40	61.7	47.1
30	84.1	73.2
25^a^	91.8	84.2
20	96.6	92.3
10	99.8	99.3
0	100	100

^a^Calculated from data reported in [26, 27]

**Table 5:** Sample size in the proposed RCT on rESWT + RP for acute HMC injury type 3b, accounting for a two-sided confidence interval of 95% and a power of 0.8. The percentage of patients with treatment success when treated with rESWT + RP was set at 75 based on own experience (unpublished data)

Percent of patients with treatment success when treated with sham-rESWT + RP (%)	Sample size of both groups (rESWT + RP, sham-rESWT + RP) according to…		
	[49]	[50]	[50] with continuity correction
99.9	28	27	35
90	99	98	111
80	1095	1094	1134
70	1221	1220	1259
60	154	152	165
50	59	58	66
40	32	31	36
30	20	19	23
25^a^	16	15	19
20	13	12	16
10	10	8	11

^a^Calculated from data reported in [26, 27]

In summary, the proposed study would have a power of less than 0.8 in finding a difference in treatment success (possibility to return to play with the criteria established in [48] fulfilled) between rESWT + RP and sham-rESWT + RP for treating acute HMC injury type 3b if the percentage of patients with treatment success when treated with sham-rESWT + RP would be higher than 30%. This, however, is not to be expected considering the aforementioned data published in [26, 27]. This reinforces the validity of the protocol of this study for testing efficacy and safety of rESWT + RP using the Swiss DolorClast for acute HMC injury type 3b.

### Follow-up and statistical analysis

Follow-up will be the same for all study patients. The design of this study guarantees that there will be full compliance with the allocated treatment and, thus, no contamination of one group.

The patient’s age, gender, body mass index, sport that is practiced, position in the field (such as attack or defense in case of soccer; if applicable), and the sporting gesture that caused the injury are potential confounding factors when treating acute HMC injury type 3b with rESWT. Normal distribution of the patients’ age and the body mass index will be tested using the D’Agostino-Pearson omnibus test. In case of passing the normality test, we will report mean and standard error of the mean of these variables; otherwise, we will report inter-quartile ranges of these variables. Comparison between groups will be performed with Student’s *t* test in case of passing the normality test or the nonparametric Mann-Whitney test in case of not passing the normality test.

The primary clinical outcome will be the individual time (days) necessary to return to play. The primary clinical outcome will return a single data point (number of days) for each patient. Time of return to play is not normally distributed data. Accordingly, we will report inter-quartile ranges of this variable. Comparison between groups will be performed using the nonparametric Mann-Whitney test.

One secondary clinical outcome will be assessment of patient’s satisfaction at 6 months after inclusion into this study (using a scale ranging from 0 (maximum dissatisfaction) to 10 (maximum satisfaction)). This secondary clinical outcome will return a single data point (on a scale ranging from 0 to 10) for each patient, which is not normally distributed data. Accordingly, we will report inter-quartile ranges of this variable. Comparison between groups will be performed using the nonparametric Mann-Whitney test.

Another secondary clinical outcome will be presence or absence of reinjury during a time period of 6 months after inclusion into the study. This secondary clinical outcome will return a single data point (“yes” or “no”) for each patient, which is not normally distributed data. Accordingly, we will report absolute and relative numbers of “yes” and “no” of this variable. Comparison between groups will be performed using Fisher’s exact test.

The probability value of less than 0.05 (*p* value < 0.05) will be considered as statistically significant [51]. All calculations will be performed using GraphPad Prism (version 5.00 for Windows, GraphPad Software, San Diego, CA, USA).

All main conclusions of the study will be based on analyses of intention to treat rather than analyses of treatment. Note that there are various available methods for handling missing data in clinical trials [52]. In case of missing data (i.e., in case a patient will withdraw or will be lost during the treatment or the follow-up periods), we will determine the most appropriate method for performing analyses of intention to treat. After randomization and the first rESWT or sham-rESWT, no patient will be replaced.

All efforts will be made to keep the proportion of patients lost to follow-up too small to affect the main findings of this study. Patient-centered care throughout this study will ensure that no patients will be lost to follow-up, or the number of patients lost to follow-up will be so small that findings would be unaffected by their inclusion. We will report actual probability values for all outcomes except where probability values less than 0.001 are found. We will avoid any retrospective unplanned subgroup analysis and, thus, “data dredging.”

## Discussion

Radial ESWT is being increasingly used for tendon and other pathologies of the musculoskeletal system. However, a randomized, sham-controlled clinical trial on ESWT for acute HMC injury type 3b in athletes has not yet been performed. Current evidence suggests that ESWT may accelerate regeneration after acute skeletal muscle injury [30], which is in line with our clinical experience using rESWT. We hypothesize that the results of this study will be of major interest, which is because of the abundant use of rESWT as well as the need for developing novel treatment strategies for acute HMC injury type 3b in athletes [26]. However, it should be mentioned that our study has a number of limitations as outlined in the following.

First, only a single rESWT device (Swiss DolorClast with EvoBlue handpiece; Electro Medical Systems) will be used. In this regard, it should be kept in mind that the majority of clinical trials on rESWT that are listed in the PEDro database [37, 38] were performed with the Swiss DolorClast [34]. Furthermore, except of the Power + handpiece of the Swiss DolorClast, the Evo Blue handpiece was the most powerful handpiece in a comparative study measuring cavitation generated with rESWs [53] (the relevance of cavitation in mediating molecular and cellular mechanisms of ESWT on the musculoskeletal system was discussed in [43, 53–55]).

Second, we will not apply MRI but ultrasonography (US) in diagnosis of acute HMC injury type 3b. However, it has been demonstrated that both MRI and US provide detailed information about the HMC with respect to localization and characterization of injury [12–14]. Furthermore, clinical, MRI, and US determinants were established in the literature that have an effect on the time to return to play after HMC injury in athletes [16]. On the other hand, it is important to realize that for an individual HMC injury, none of these MRI and US determinants show a direct correlation with the time to return to play [14, 15].

Third, our study does not include a sham control group, and/or a group that only receives rESWT, and/or a group that only gets a specific rehabilitation program (RP). Rather, all the patients will get a specific RP in addition to rESWT or sham-rESWT, respectively. Providing a specific RP is the way most therapists treat acute HMC injury type 3b in athletes today [12, 26, 27], and including sham-rESWT in the present study will make sure that any difference in results between the groups is due to the rESWT treatment, and not the placebo effect. Inclusion of the specific RP in the study protocol appears mandatory because rESWT for acute HMC injury type 3b in athletes does only make sense in case the combination of rESWT and RP will result in statistically significantly better therapeutic outcome (i.e., reduced time to return to play) than the combination of sham-rESWT and RP. In this regard, it is critical to note that in an early study on treatment of acute muscle strain injuries (that were experimentally induced in rats) with platelet rich-plasma (PRP) but without RP, a shortened recovery time was observed compared to treatment with platelet-poor plasma [56], but this could not be reproduced when treating acute HMC injury type 3b in athletes with a combination of PRP and RP or a combination of injections of saline and RP [26].

In conclusion, a positive result of this study may change current practice, while a negative result (i.e., no statistically significant difference between the groups) will indicate that the use of rESWT for acute HMC injury type 3b in athletes should not be recommended.

## Data Availability

The datasets used and/or analyzed during the current study are available from the corresponding author on reasonable request.

## Abbreviations

A: Age
BMI: Body mass index
D: Day
ESWL: Extracorporeal shock wave lithotripsy
ESWT: Extracorporeal shock wave therapy
fESWT: Focused extracorporeal shock wave therapy
G: Gender
HMC: Hamstring muscle complex
LHBF: Long head of the biceps femoris
M: Month
MRI: Magnetic resonance imaging
P: Position in the field
PRP: Platelet-rich plasma
RCTs: Randomized controlled trials
rESWs: Radial extracorporeal shock waves
rESWT: Radial extracorporeal shock wave therapy
RP: Rehabilitation program
S: Sport that is practiced
SG: Sporting gesture that caused an injury
US: Ultrasonography
VAS: Visual analogue scale
